# Adenosine 5′-triphosphate (ATP) supplements are not orally bioavailable: a randomized, placebo-controlled cross-over trial in healthy humans

**DOI:** 10.1186/1550-2783-9-16

**Published:** 2012-04-17

**Authors:** Ilja CW Arts, Erik JCM Coolen, Martijn JL Bours, Nathalie Huyghebaert, Martien A Cohen Stuart, Aalt Bast, Pieter C Dagnelie

**Affiliations:** 1 Department of Epidemiology, Maastricht University, P.O. Box 616, Maastricht, MD, 6200, The Netherlands; 2 Department of Toxicology, Maastricht University, P.O. Box 616, Maastricht, MD, 6200, The Netherlands; 3 Laboratory of Pharmaceutical Technology, Faculty of Pharmaceutical Sciences, Ghent University, Harelbekestraat 72, Ghent, B-9000, Belgium; 4 Department of Agrotechnology and Food Sciences, Laboratory of Physical Chemistry and Colloid Science, Wageningen University, P.O. Box 8038, Wageningen, EK, 6700, The Netherlands

**Keywords:** ATP, Metabolism, Nutritional supplement, Bioavailability, Gastrointestinal transit, Multi-particulate supplement

## Abstract

**Background:**

Nutritional supplements designed to increase adenosine 5′-triphosphate (ATP) concentrations are commonly used by athletes as ergogenic aids. ATP is the primary source of energy for the cells, and supplementation may enhance the ability to maintain high ATP turnover during high-intensity exercise. Oral ATP supplements have beneficial effects in some but not all studies examining physical performance. One of the remaining questions is whether orally administered ATP is bioavailable. We investigated whether acute supplementation with oral ATP administered as enteric-coated pellets led to increased concentrations of ATP or its metabolites in the circulation.

**Methods:**

Eight healthy volunteers participated in a cross-over study. Participants were given in random order single doses of 5000 mg ATP or placebo. To prevent degradation of ATP in the acidic environment of the stomach, the supplement was administered via two types of pH-sensitive, enteric-coated pellets (targeted at release in the proximal or distal small intestine), or via a naso-duodenal tube. Blood ATP and metabolite concentrations were monitored by HPLC for 4.5 h (naso-duodenal tube) or 7 h (pellets) post-administration. Areas under the concentration vs. time curve were calculated and compared by paired-samples t-tests.

**Results:**

ATP concentrations in blood did not increase after ATP supplementation via enteric-coated pellets or naso-duodenal tube. In contrast, concentrations of the final catabolic product of ATP, uric acid, were significantly increased compared to placebo by ~50% after administration via proximal-release pellets (P = 0.003) and naso-duodenal tube (P = 0.001), but not after administration via distal-release pellets.

**Conclusions:**

A single dose of orally administered ATP is not bioavailable, and this may explain why several studies did not find ergogenic effects of oral ATP supplementation. On the other hand, increases in uric acid after release of ATP in the proximal part of the small intestine suggest that ATP or one of its metabolites is absorbed and metabolized. Uric acid itself may have ergogenic effects, but this needs further study. Also, more studies are needed to determine whether chronic administration of ATP will enhance its oral bioavailability.

## Background

Nutritional supplements designed to increase adenosine 5′-triphosphate (ATP) concentrations are commonly used by athletes as ergogenic aids. ATP is the primary source of energy for the cells, and supplementation may enhance the ability to maintain high ATP turnover during high-intensity exercise. ATP is also released from cells to act as a local regulator of neurotransmission, inflammation, and nociception via interaction with purinergic receptors [1,2]. ATP is present in substantial concentrations in a number of foods (e.g. meat, soy, mushrooms) [3] and in breast milk [4,5]. Furthermore, capsules containing ATP are currently registered in France for the treatment of low back pain of muscular origin, and supplements containing ATP are marketed on the internet for various purposes including the restoration of energy.

Oral ATP supplements have beneficial effects in some but not all studies examining physical performance. In an experimental study by Jordan *et al.*[6], three groups of nine healthy men received ATP (150 or 225 mg) or placebo for 14 days. Physical performance and muscular strength were positively affected. Another study investigated the effects of supplementation with an ATP-containing registered drug for 30 days (Atépadène®, 90 mg daily) [7,8]. The questionnaire-based outcome indicated that it provided benefit to patients with subacute low back pain. In contrast to these beneficial findings, Herda et al. [9] found no improvements in muscle strength, power output, or endurance after supplementation of 24 healthy men with a commercially available treatment intended to increase ATP. The authors suggested that the lack of an effect in this double-blind, placebo-controlled crossover trial, might be caused by breakdown of ATP in the gastrointestinal tract. Because they did not collect blood samples from the participants, the authors could not verify whether ATP concentrations in the blood circulation had been altered as a result of supplementation [9].

Evidence on the oral availability of ATP supplements is limited. In the study by Jordan et al. [6], no changes in whole blood and plasma ATP concentrations were detected, but the dosages administered were modest (225 mg or less). Animal studies reporting alterations in cardiac, vascular and pulmonary function after 30 days of oral ATP supplementation, also found no increases in systemic concentrations of plasma or erythrocyte ATP [10,11]. However, the concentration of ATP in plasma taken from the portal vein of rats increased rapidly up to a 1000-fold after instillation of ATP in de small intestine [11]. The identification of a number of nucleoside transporters in the small intestine further suggested that orally administered ATP may be absorbed and utilized by the human body [12].

We have previously shown that ATP is bioavailable after intravenous administration in humans [13]. ATP concentrations in erythrocytes increased in a dose-dependent manner by ~60% after 24 h of continuous infusion. We now report the results of a randomized, placebo-controlled, cross-over trial in 8 healthy humans, designed to assess the oral bioavailability of an ATP nutritional supplement. The ATP was administered as a single dose that was high enough to enable its detection in whole blood (5000 mg). Furthermore, an acid-resistant enteric coating of the multi-particulate supplement was used to prevent the degradation of ATP in the acidic environment of the stomach. As a comparison, ATP was also directly instilled in the small intestine via a naso-duodenal tube.

## Methods

### Study design

Subjects were examined on five occasions according to a cross-over design. Two types of enteric coated pH-sensitive multi-particulate supplements (from now on referred to as pellets) were tested, one targeting the proximal part of the small intestine, and one targeting the distal part. On days 0, 7, and 14, subjects received the following supplements in random order: 5000 mg ATP as proximal-release pellets, 5000 mg ATP as distal-release pellets, or placebo proximal-release pellets. The pellets were ingested with approximately 200 mL water acidified to pH < 5 with citric acid. On days 21 and 28, subjects received in random order 5000 mg ATP dissolved in 100 mL water (30 ± 4°C), or water only (placebo), administered through a naso-duodenal tube. The tube was inserted through the subjects’ nostril and placed in the stomach. To promote movement of the tube through the pylorus into the duodenum, subjects were asked to lay down on their right side. To verify the tube’s position (either stomach or duodenum), gastro-intestinal juice samples were taken by a syringe and tested for their pH and color. Once pH was above 5 (±180 min after insertion of the tube), and color was yellow, administration started and the tube was removed 10 min later. The study was approved by the Medical Ethics Committee of Maastricht University Medical Centre. The study was carried out according to the Helsinki Declaration for human experiments.

### Study population

Male and female subjects (18–60 years) received oral and written information about the protocol and possible risks before signing informed consent. Exclusion criteria were a history of lung, heart, intestinal, stomach or liver disease, use of prescription medication, smoking, drug use, dietary restrictions, and pregnancy. Subjects abstained from products containing alcohol or caffeine and from purine-rich foods, such as game, offal, sardines, anchovies and alcohol-free beer for two days before each test day. Subjects fasted from 10 p.m. the previous day until the end of the test day (4 p.m.), and refrained from any vigorous physical activity starting 24 h before each test day. Subjects were allowed to drink water starting 30 min after ATP or placebo administration.

## Materials

ATP disodium salt was purchased from Pharma Waldhof GmbH, Düsseldorf, Germany. Adenosine 5′-diphosphate (ADP) disodium salt, adenosine 5′-monophosphate (AMP) sodium salt, adenine, inosine, hypoxanthine, uric acid and nitric acid were purchased from Sigma Chemical Co., St. Louis, USA. Adenosine and lithium carbonate (Li_2_CO_3_) were obtained from Fagron BV., Uitgeest, The Netherlands. Perchloric acid (PCA) 70% solution in water was purchased from Sigma-Aldrich, Steinheim, Germany. KOH, KH_2_PO_4_, K_2_CO_3_, K_2_HPO_3_*3H_2_O and NaOH were obtained from Merck, Darmstadt, Germany and 0.9% saline from Braun, Melsungen, Germany. Bengmark-type naso-duodenal tubes were from Flocare, Zoetermeer, The Netherlands. NH_4_NO_3_ was obtained from Fluka, Steinheim, Germany. Trichloroacetic acid (TCA) 20% solution in water was from Serva, Heidelberg, Germany. Citric acid suitable for human consumption was obtained from the pharmacy of Maastricht University Medical Centre.

### Production of pellets

ATP pellets were produced at Ghent University, Faculty of Pharmaceutical Science, Belgium as described by Huyghebaert et al. [14], with minor modifications to obtain an ATP concentration of >40% (wt:wt) after coating. Placebo pellets were produced in the same manner, but without ATP. To verify the timing of intestinal release, Li_2_CO_3_ (60 mg per administration) was added to the pellets. The proximal-release pellets were coated with 30% Eudragit® L30D-55 (ATP or placebo pellets), and the distal-release pellets (ATP only) were coated with 15% Eudragit® FS 30 D (Röhm Pharma, Darmstadt, Germany), mixed with anionic copolymers of methacrylic acid and ethylacrylate (1:1). After coating, the pellets were cured overnight at room temperature at 60% (proximal-release pellets) or 20% (distal-release pellets) humidity, packed in aluminum foil sachets (VaporFlex®, LPS, NJ, USA), sealed at their respective humidity and stored at room temperature. Pellets were used within 3 months after production.

### Dissolution testing

To test whether the coating of the pellets was adequate, a dissolution test (*n* = 3 for each type of coating) was performed using the reciprocating cylinder method (USP apparatus 3 from Bio-Dis, VanKel, NJ, USA) at a dip rate of 21 dips per minute using 3 g pellets per vessel (250 mL) with two consecutive media: 0.1 N HCl (37°C), and a 0.2 M KH_2_PO_4_ buffer (37°C) with a pH that was adjusted to 6.5 for the proximal-release pellets, and pH 7.4 for the distal-release pellets. Samples were collected after 2 h in HCl and after 2, 5, 10, 20, 30 and 60 min in buffer as described in Huyghebaert et al. [14]. ATP and metabolite concentrations were measured by HPLC separation and UV-analysis as previously described [15].

### Sample collection during the intervention

Venous blood was collected from the antecubital vein by a 20 gauge intravenous catheter (Terumo-Europe NV, Leuven, Belgium), connected to a three-way stopcock (Discofix®, Braun Melsungen AG, Melsungen, Germany). Blood was collected into 4 mL EDTA tubes (Venosafe, Terumo-Europe NV) by inserting a 21 gauge multisample needle (Venoject Quick Fit, Terumo-Europe NV) into the membrane of a closing cone (IN-Stopper, Braun Melsungen AG) that was attached directly to the stopcock. The anticoagulant EDTA inhibits the extracellular hydrolysis of ATP by Ca^2+^- and Mg^2+^-activated enzymes such as plasma membrane-bound CD39 [16]. To avoid clotting after each blood collection, approximately 1.5 mL of heparinized (50 I.E./mL) 0.9% saline was used to rinse the blood collection set-up. It was removed before the next blood collection.

Three baseline blood samples were collected at 30, 20 and 10 min before administration. Starting 30 min after pellet administration or 15 min after naso-duodenal administration, blood samples were collected every 15 min. Between 210 and 420 min (pellets) or 270 min (naso-duodenal tube) after administration, samples were collected every 30 min. Total volume collected per day was 92 mL.

After blood collection, the tubes were inverted three times and put on ice. Five hundred μL of blood was added to 500 μL ice-cold PCA (8% wt:v), vortex-mixed and frozen in liquid nitrogen. Untreated plasma samples (centrifugation at 3000 rpm, 10 min, 4°C) were collected for assessment of lithium release from the pellets. All samples were stored at -80°C awaiting analysis.

### ATP measurement in whole blood by HPLC

Equipment, sample preparation and measurement conditions have been previously described and validated [15]. Briefly, after thawing, the protein fraction was precipitated (12,000 g, 10 min, 4°C) and 40 μL 2 M K_2_CO_3_ in 6 M KOH was added to 650 μL supernatant to neutralize the pH. The resulting insoluble perchlorate was removed by centrifugation (12,000 g, 10 min, 4°C), and 40 μL supernatant was mixed with 160 μL 0.05 M phosphate buffer pH 6.0 in HPLC vials.

### Lithium measurement in plasma

To investigate the timing of pellet disintegration, plasma concentrations of the lithium marker were measured using a modified Trapp protocol [17]. Following thawing on ice, 50 μL plasma was vortex-mixed with 10 μL trichloroacetic acid (20% v:v) and centrifuged (14,000 rpm, 10 min) to precipitate the proteins. The supernatant was diluted 20 times in 0.1 M nitric acid, which also served as the blank. Two replicate measurements per sample were performed on a SpectrAA 400 graphite tube atomic absorption spectrophotometer (AAS) (Varian, Palo Alto, CA, USA) with a lithium hollow-cathode lamp, operated at 5 mA and a 1.0 nm slit. Peak height measurements at 670.8 nm wavelength were compared with values for standards of known concentrations (ranging from 2 to 10 ng/mL). Initially, 20 μL sample and 5 μL modifier solution (1.2 M NH_4_NO_3_) were injected into the top hole of the graphite tube. Then, fluids were evaporated at 95°C for 40 s and at 120°C for 10 s. The ash time was 15 s at 700°C, followed by atomization at 2300°C with a 3 s read time. If the obtained signal exceeded the standard concentration range (0–10 ng/mL), samples were diluted with blank and measured again.

### Statistical analysis

The area under the concentration vs. time curve (AUC) was calculated using the linear trapezoidal rule from time zero until the last time point of sampling *t* (AUC_0-*t*_). *C*_*min*_ and *C*_*max*_ were defined as the minimum and maximum observed concentrations, respectively. *t*_*max*_ was the time at which *C*_*max*_ was reached. AUC of the five conditions were compared and analyzed by paired-samples t-tests. A *P-value* < 0.05 was considered statistically significant. Analyses were performed with the SPSS software package version 16.0 for Windows.

## Results

Eight subjects (6 females and 2 males, aged 26.9 ± 5.9 years (mean ± SD), weighing 70 ± 4.3 kg, with a BMI of 23.6 ± 1.3) completed the trial. No adverse events were observed with both types of administration (i.e. pellets, solution).

**Figure 1:**
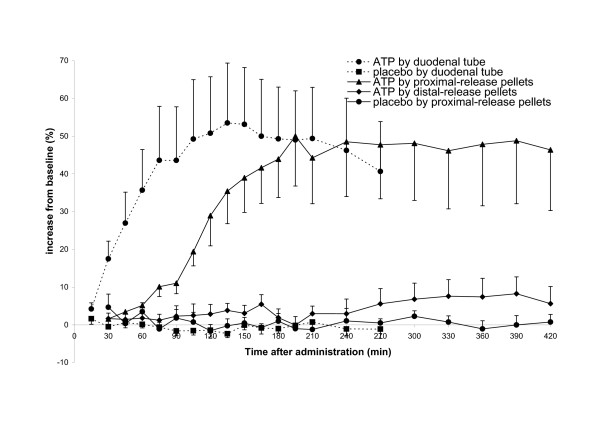
**Uric acid concentrations in healthy volunteers after oral ATP or placebo supplementation.** A single dose of 5000 mg ATP or placebo was administered via proximal-release pellets, distal-release pellets, or naso-duodenal tube. Data are presented as percentage increase from the mean of three blood samples taken before administration. Values are means ± SEM, *n* = 8.

HPLC analysis of the whole blood showed that ATP concentrations were stable over time, and that there were no statistically significant differences between placebo and ATP supplements for any type of administration (data not shown). Of the other metabolites (ADP, AMP, adenosine, adenine, inosine, hypoxanthine, and uric acid), only uric acid concentrations changed in response to supplement administration (Figure 1). Compared to placebo, the uric acid AUC increased significantly when ATP was administered by proximal-release pellets (*P* = 0.003) or by naso-duodenal tube (*P* = 0.001). Administration of ATP by distal-release pellets did not lead to a significantly increased uric acid AUC, compared to placebo. The peak uric acid concentrations (C_*max*_) were 36% higher (0.28 ± 0.02 mmol/L) for proximal-release pellets compared to distal-release pellets (0.21 ± 0.01 mmol/L), but 6% lower compared to the administration via naso-duodenal tube (0.30 ± 0.02 mmol/L) (Figure 1 and statistics in Table 1). The mean time to peak uric acid concentration (t_max_) was shorter for naso-duodenal tube administration (t_max_ ranged from 75 to 195 min with mean ± SD 135 ± 15 min) as compared to the pellet administration (t_max_ ranged from 150 to 390 min with mean ± SD 234 ± 32 min). An overview of the inter-subject variability in uric acid concentrations following administration of ATP (tube and pellets) is presented in Additional file 1: Figure S1.

**Table 1:** Pharmacokinetic parameters for uric acid and lithium after oral administration of ATP

***Mode of administration (time period)***	***AUC uric acid mmol.min/L***	***C***_***max***_***mmol/L (range)***	***t***_***max***_***min (range)***	***AUC Lithium mmol.min***
Naso-duodenal tube				
ATP (270 min)	19.6 ± 4.4 ^a,b,c^	0.31 ± 0.03	135	n.a.
		(0.23-0.38)	(105–240)	
Placebo (270 min)	−0.4 ± 0.4	0.21 ± 0.03	n.a.	n.a.
		(0.15-0.33)		
Proximal-release pellets				
ATP (270 min)	16.1 ± 3.0	n.a.	n.a.	n.a.
Placebo (270 min)	0.8 ± 0.9	n.a.	n.a.	n.a.
ATP (420 min)	25.4 ± 5.7 ^d,e^	0.30 ± 0.03	240	65174 ± 7985 ^f^
		(0.21-0.41)	(165–390)	
Placebo (420 min)	0.9 ± 1.1	0.20 ± 0.02	n.a.	117914 ± 15021 ^f^
		(0.16-0.31)		
Distal-release pellets				
ATP (270 min)	1.7 ± 1.1	n.a.	n.a.	n.a.
ATP (420 min)	3.2 ± 1.4	0.22 ± 0.02	390	12575 ± 2832 ^f^
		(0.17-0.34)	(105–420)	

Values are group means ± SEM, *n* = 8 per formulation, P-values are based on paired-samples t-tests. N.a. = not available.

^a^ Different from naso-duodenal tube placebo (*P* = 0.002), ^b^ Different from ATP distal-release pellets 270 min (*P* = 0.007), ^c^ Different from proximal-release placebo pellets 270 min (*P* = 0.007) ^d^ Different from ATP distal release pellets 420 min (*P* = 0.005), ^e^ Different from proximal-release placebo pellets (*P* = 0.005), ^f^ Different from each other (*P* < 0.001).

**Figure 2:**
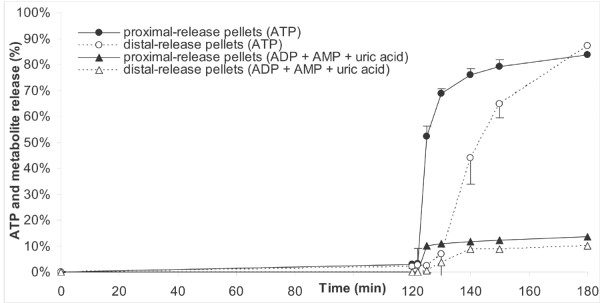
**Release of ATP and metabolites from enteric coated supplement after dissolution testing.** Release of ATP and its metabolites as a percentage of the release at 180 min for proximal-release pellets (closed symbols) and distal-release pellets (open symbols), after 120 min in 0.1 N HCl, and subsequently 60 min in buffer solutions with either pH 6.5 (proximal-release pellets) or 7.4 (distal-release pellets). Data were obtained by the reciprocating cylinder method (USP apparatus 3). Values are means ± SEM, *n* = 3.

To verify whether the coating of the pellets had been adequate, they were tested in a dissolution experiment. Figure 2 shows the percentage of ATP that was released from the pellets, either as ATP or as any of its metabolites. After staying for 120 min in 0.1 N HCl, less than 5% ATP (5.0 ± 0.6% for the proximal-release pellets and 3.4 ± 0.4% for the distal-release pellets) was released from the pellets. Subsequent rapid changing of the buffer solutions to pH 6.5 or 7.4 for 60 min caused a release of 50% of the remaining ATP within 5 min (proximal-release pellets) or 25 min (distal-release pellets), which increased to >80% after 60 min. ATP was partially broken down to ADP (8.6% for proximal-release pellets, 7.0% for distal-release pellets), AMP (1.0 and 0.7%, respectively), and uric acid (4.0 and 2.5%, respectively).

**Figure 3:**
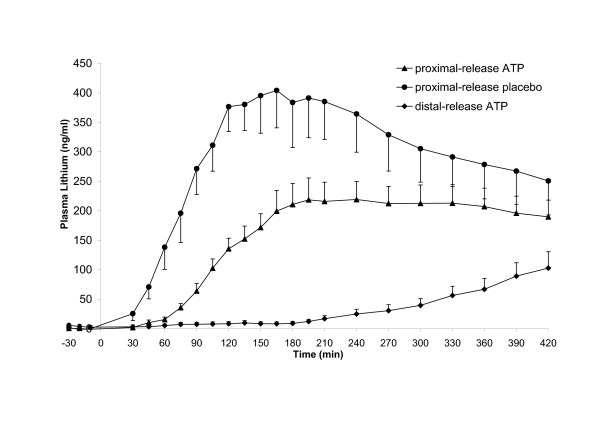
**Plasma lithium concentrations in healthy volunteers after administration of supplement containing 60 mg Li**_**2**_**CO**_**3**_**.** A single dose of 5000 mg ATP or placebo with 60 mg Li_2_CO_3_ was administered via proximal-release pellets or distal-release pellets. Values are means ± SEM, *n* = 8.

Finally, to investigate whether the timing of pellet disintegration in the gastrointestinal tract had been as expected, plasma lithium concentrations were determined in samples collected for 7 h after administration of the coated pellets (Figure 3). The three types of pellets had different release profiles, as was quantified by measuring the AUC (Table 1). Comparison of the AUC of the two types of ATP-containing pellets revealed that the proximal-release pellets caused a significantly higher increase in plasma lithium than the distal-release pellets (*P* = 0.001) (Figure 3). Further comparison of the proximal-release pellets with or without ATP, showed that the lithium AUC was significantly lower in the ATP-containing pellets than in the placebo-containing ones (*P* = 0.001). Individual plasma lithium concentrations are depicted in Additional file 2: Figure S2. Lithium C_*max*_ for the proximal release pellets was reached between 135 and 210 min after administration at a mean concentration of 404 ng/mL for the placebo pellets and 200 ng/mL for the ATP pellets. The highest plasma lithium concentration (717 ng/mL) was measured in a volunteer receiving placebo proximal-release pellets. The distal-release pellets, on the other hand, showed a delayed and lower release profile, with lithium concentrations starting to rise only approximately 240 min after administration, while a maximum concentration of 103 ng/mL was reached at the final measurement.

## Discussion

The aim of this study was to determine the oral bioavailability of ATP after targeted delivery to the small intestine using two types of enteric coated pH-sensitive multi-particulate supplements. As a comparison, ATP was also directly instilled in the small intestine via a naso-duodenal tube. Although the ATP dosage administered in our study (5000 mg, or 55.6 - 83.3 mg/kg body weight) exceeded those of most other oral administration studies, we observed no changes in whole blood ATP concentrations. Recommended dosages to ‘increase your energy’ for ATP supplements marketed on the internet usually range from 100–250 mg per day, which is considerably lower that the dosage we tested. The only other human study that we know of that measured ATP after oral administration of either 150 mg or 225 mg ATP as enteric coated beadlets, also found no increase in plasma and whole blood ATP concentrations [6]. Kichenin *et al*. orally administered ATP in dosages up to 20 mg/kg per day to rabbits and up to 10 mg/kg per day to rats [10,11]. No increases in systemic plasma or erythrocyte ATP concentrations were observed. However, the concentration of ATP in plasma taken from the portal vein of rats increased rapidly up to a 1000-fold after direct instillation of ATP in the small intestine. In humans it is not possible to collect portal vein blood without performing very invasive procedures, and we could therefore not determine this is our study. Intravenous ATP administration in humans ranging in dosage from 36 to 108 mg/kg per day [13,18,19] did lead to substantial increases in ATP concentration in the systemic circulation of up to 60% above baseline. Of the ATP metabolites considered, only uric acid concentrations increased significantly after administration of the proximal-release pellets and of the naso-duodenal tube, but not of the distal-release pellets.

When ATP is released into the small intestine, ecto-nucleotidase triphosphatase diphosphohydrolases present on the luminal side of intestinal enterocytes dephosphorylate ATP via ADP to AMP [20], after which ecto-5′-nucleotidase (CD73) degrades AMP to adenosine [21]. In mice, the terminal ileum is the site in the intestine with the lowest ATPase activity [22]. Although information on the human intestine is limited, this may explain the difference in plasma uric acid concentrations after ingesting the proximal or distal-release pellets. Concentrative (CNT) and equilibrative (ENT) nucleoside transporters are able to transport nucleosides into the intestinal enterocytes and to the capillary bed of the intestinal villi. CNTs exhibit a proximal-distal gradient with highest transport activities present in the jejunum [23]. Finally, adenosine is taken up by the erythrocytes through ENTs in the erythrocyte membrane [24]. *In vivo* studies in animals and humans indicated that inside the erythrocytes adenosine can be used for the synthesis of ATP [19]. In our study, neither ATP nor adenosine concentrations were increased, suggesting that instead of being used for ATP synthesis in the erythrocytes, orally administered ATP is degraded to uric acid by xanthine oxidase, an enzyme which is expressed mainly in the liver and in endothelial cells of blood vessels [25]. Assuming that uric acid is primarily present in the extracellular fluid (the volume of which is approximately 22% of body weight), that the 5000 mg ATP is completely broken down to 9.06 mmol uric acid, and that there is no loss of uric acid due to excretion, the estimated ‘bioavailability’ of ATP (defined as the observed uric acid increase as a percentage of the theoretical maximum) was 16.6 ± 2.3% for the naso-duodenal tube, 14.9 ± 2.5% for the proximal-release pellets and 3.2 ± 0.6% for the distal-release pellets.

In our study, the increase in plasma uric acid concentration was similar for the proximal-release pellets and the naso-duodenal tube, indicating complete release of ATP from the pellets. The delay in uric acid increase of about 1 h following proximal-release pellet administration compared to naso-duodenal tube administration is probably a combined effect of gastric residence time and the time needed for dissolution of the coating of the pellets. We used enteric pH-sensitive coated pellets because they were previously successfully used for the targeted delivery of various compounds [26-28]. The pH-sensitive Eudragit® polymer coating provided sufficient gastroresistance, as unwanted *in vitro* release of ATP from the pellets was within the limits set by the USP (i.e. <10% drug release in 2 h in 0.1 N HCl) [29]. *In vivo*, the intestinal pH and transit times are the main factors determining the location where each type of coating releases its contents. The duodenum has a pH of 6.4 with a mean transit time to the jejunum of 30 min, while in the ileum, the pH rises to 7.4 with a transit time to the colon for pellet dosage forms in fasted individuals of approximately 3 ± 1 h (mean ± SD) [30-32]. The modest rise in uric acid concentration after ingestion of the distal-release pellets may be partly caused by incomplete release in the small intestine, in combination with the limited uptake of ATP once it has entered the colon [33]. Timely release of the contents of the pellets was confirmed by using lithium as a marker. As expected from earlier studies in which lithium was used as a marker [34], the lithium dosage administered to the subjects was safe; the highest plasma lithium concentration amounted to only 17% of the lower therapeutical range advised for patients with bipolar disease [35]. Remarkably, higher lithium concentrations were reached after administration of the placebo pellets compared to ATP pellets. Possibly, the higher content of carboxymethylcellulose (CMC), which promotes pellet disintegration by expanding upon contact with water, in the placebo pellets (nearly 100%), compared to the ATP pellets (nearly 50%), resulted in a quicker release of lithium and hence the higher plasma concentration. Another possibility is that the negative charges on the CMC molecule, which promote its exposure to water, are shielded by the sodium-ions in the ATP pellets, thus slowing the swelling of CMC in the pellets and thereby the release of their contents.

What may be the consequences of increased plasma uric acid concentrations obtained by orally administering ATP? On the one hand, hyperuricemia is a risk factor for gout and is associated with hypertension [36-39]. The highest individual uric acid concentration (405 μmol/L) we observed, is within the range reported for male non-gouty individuals (179–440 μmol/L) [40]. No adverse effects were observed during the study. The short-lasting increase in uric acid concentration found in the current study is not likely to cause any symptoms of gout or hypertension, since these require a prolonged period of severe increase [41]. On the other hand, high uric acid concentrations have also been associated with beneficial health effects. Uric acid may function as an antioxidant [42,43], and epidemiological studies have shown that healthy subjects with high uric acid concentrations are at a reduced risk for developing Parkinson’s disease, a condition suspected to be instigated by oxidative damage [44,45]. Furthermore, patients with multiple sclerosis are known to have lower uric acid concentrations than healthy volunteers, and raising the uric acid concentration by pharmacological means has been the subject of recent investigation [46]. Although increasing the uric acid concentration pharmacologically using ATP pellets might have benefits for certain individuals, these have to be weighed against increased risks of gout and possibly cardiovascular disease [36,38,39].

## Conclusions

A single dose of oral ATP supplement is not bioavailable, whether administered as proximal-release or distal-release enteric coated pellets, or directly instilled in the small-intestine. This may explain why several studies did not find ergogenic effects of oral ATP supplementation. An on average 50% increase in uric acid concentration was found with the proximal-release pellets and with the naso-duodenal tube, suggesting that ATP or one of its metabolites is absorbed, but immediately metabolized before becoming available to the body. Uric acid itself may have beneficial effects, but this needs further study. Also, more studies are needed to determine whether chronic administration of ATP will enhance its oral bioavailability.

## Abbreviations

AAS, Atomic absorption spectrophotometer; ADP, Adenosine 5′-diphosphate; AMP, Adenosine 5′-monophosphate; ATP, Adenosine 5′-triphosphate; AUC, Area under the curve; CD73, Ecto-5′-nucleotidase; carboxymethylcellulose; CNT, Concentrative nucleoside transporter; ENT, Equilibrative nucleoside transporter; PCA, Perchloric acid; TCA, Trichloroacetic acid.

## Competing interests

The authors declare that they have no competing interests.

## Authors’ contributions

ICWA participated in the design and data analysis of the study, and drafted the manuscript, EJCMC carried out the human intervention study, participated in the data analysis and drafted the manuscript, MJLB participated in the design of the study and helped to draft the manuscript, NH produced the pellets and carried out the dissolution experiments, MACH participated in the design of the study and helped to draft the manuscript, AB participated in the design and conception of the study and helped to draft the manuscript, PCD conceived of the study, participated in the design and coordination of the study, and helped to draft the manuscript. All authors read and approved the final manuscript.

## Supplementary Material

Additional file 1 **Figure S1. Individual increases in plasma uric acid concentrations following supplementation with 5000 mg ATP.** ATP was administered at t = 0 as a solution through a naso-duodenal tube (**A**), proximal-release pellets (**B**), or distal-release pellets (**C**). Values represent the percentage increase from the mean baseline values that were determined in three samples collected at 30, 20 and 10 min before administration. The legend shows sex of subjects. Note the different scale of the x-axis in panel A. Click here for file

Additional file 2 **Figure S2. Individual increases in plasma lithium concentrations after administration of supplement containing 60 mg Li**_**2**_**CO**_**3**_**.** Plasma lithium concentrations (ng/ml) of 6 female and 2 male volunteers after (**A**) proximal-release pellets containing ATP, (**B**) proximal-release pellets containing placebo or (**C**) distal-release pellets containing ATP. Click here for file
