# Supplementary figures and images for: Adenosine 5′-triphosphate (ATP) supplements are not orally bioavailable: a randomized, placebo-controlled cross-over trial in healthy humans
Source: J Int Soc Sports Nutr. 2012 Apr 17;9:16. doi: 10.1186/1550-2783-9-16 (PMC3441280; doi:10.1186/1550-2783-9-16)

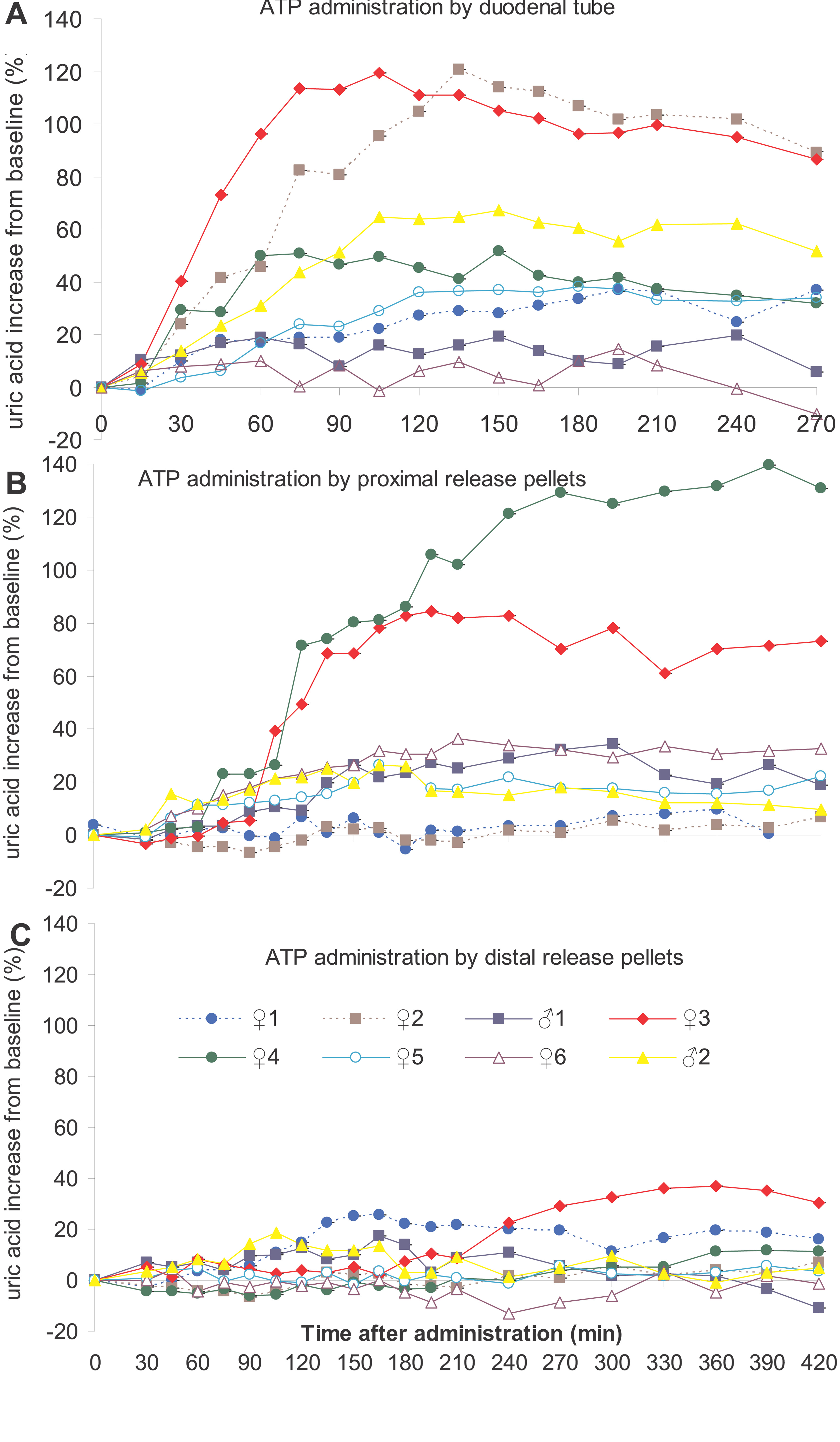

Supplement: Additional file 1 — Figure S1. Individual increases in plasma uric acid concentrations following supplementation with 5000 mg ATP. ATP was administered at t = 0 as a solution through a naso-duodenal tube (A), proximal-release pellets (B), or distal-release pellets (C). Values represent the percentage increase from the mean baseline values that were determined in three samples collected at 30, 20 and 10 min before administration. The legend shows sex of subjects. Note the different scale of the x-axis in panel A. [file 1550-2783-9-16-S1.jpeg]

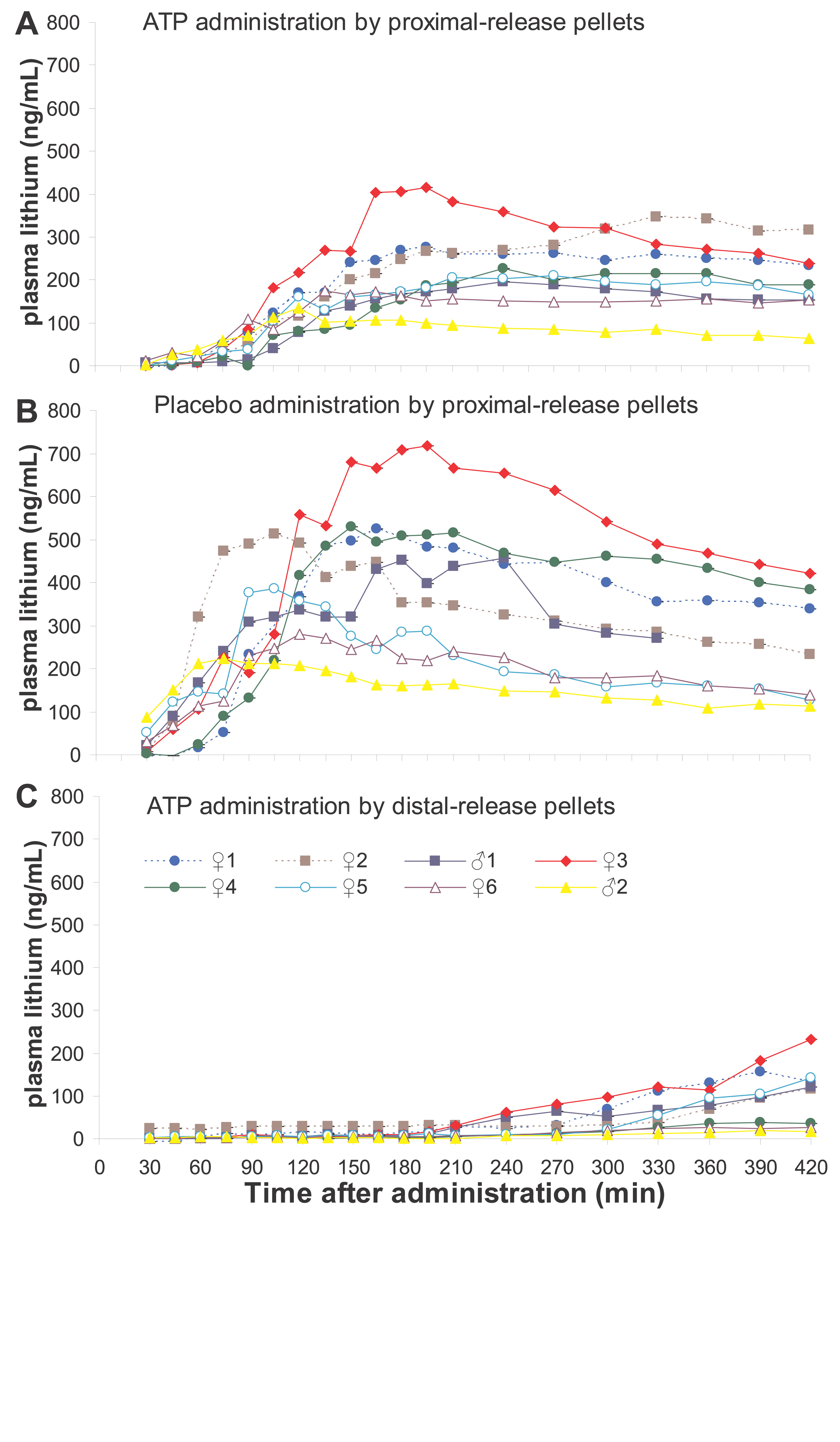

Supplement: Additional file 2 — Figure S2. Individual increases in plasma lithium concentrations after administration of supplement containing 60 mg Li2CO3. Plasma lithium concentrations (ng/ml) of 6 female and 2 male volunteers after (A) proximal-release pellets containing ATP, (B) proximal-release pellets containing placebo or (C) distal-release pellets containing ATP. [file 1550-2783-9-16-S2.jpeg]
